# Pharmacological Evaluation and Preliminary Pharmacokinetics Studies of a New Diclofenac Prodrug without Gastric Ulceration Effect

**DOI:** 10.3390/ijms131115305

**Published:** 2012-11-19

**Authors:** Jean Leandro dos Santos, Vanessa Moreira, Michel Leandro Campos, Rafael Consolin Chelucci, Karina Pereira Barbieri, Pollyana Cristina Maggio de Castro Souto, Márcio Hideki Matsubara, Catarina Teixeira, Priscila Longhin Bosquesi, Rosângela Gonçalves Peccinini, Chung Man Chin

**Affiliations:** 1 Lapdesf—Laboratory of Research and Drug Development, Drugs and Medicines Department, School of Pharmaceutical Science—UNESP Rodovia Araraquara Jaú Km. 01, 14801-902, Araraquara, SP, Brazil; E-Mails: rafaelchelucci@hotmail.com (R.C.C.); kakabarbieri85@yahoo.com.br (K.P.B.); bosquesi@fcfar.unesp.br (P.L.B.); 2 Pharmacology Laboratory, Inflammation unit, Butantan Institute, Vital Brazil Avenue 1500, 05503-900, São Paulo, Brazil; E-Mails: vanessam@butanta.gov.br (V.M.); pollyanasouto@usp.br (P.C.M.C.S.); marciomatsubara@butantan.gov.br (M.H.M.); catarinateixeira@butantan.gov.br (C.T.); 3 Natural Active Principles and Toxicology Department, School of Pharmaceutical Science—UNESP Rodovia Araraquara Jaú Km. 01, 14801-902, Araraquara, SP, Brazil; E-Mails: michelcampos7@yahoo.com.br (M.L.C.); peccinin@fcfar.unesp.br (R.G.P.)

**Keywords:** NSAIDs, anti-inflammatory, diclofenac, prodrugs, molecular modification, lactam, COX-inhibitor, bioconversion, chronic inflammation

## Abstract

Long-term nonsteroidal anti-inflammatory drugs (NSAIDs) therapy has been associated with several adverse effects such as gastric ulceration and cardiovascular events. Among the molecular modifications strategies, the prodrug approach is a useful tool to discover new safe NSAIDs. The 1-(2,6-dichlorophenyl)indolin-2-one is a diclofenac prodrug which demonstrated relevant anti-inflammatory properties without gastro ulceration effect. In addition, the prodrug decreases PGE_2_ levels, COX-2 expression and cellular influx into peritoneal cavity induced by carrageenan treatment. Preliminary pharmacokinetic studies have shown *in vivo* bioconversion of prodrug to diclofenac. This prodrug is a new nonulcerogenic NSAID useful to treat inflammatory events by long-term therapy.

## 1. Introduction

Nonsteroidal anti-inflammatory drugs (NSAIDs) are one of the most widely used classes of medications worldwide that are generally used to treat pain, fever and inflammation [1]. Other NSAIDs’ use as prophylactic treatment have been investigated in diseases with chronic inflammation characteristics such as Alzheimer’s disease, atherosclerosis, cancer, thrombosis, among others [2–5]. The best-known mechanism of action of NSAIDs is associated with inhibition of prostaglandin (PG) and thromboxane (TX) production by interaction with cyclooxygenase (COX-1 and COX-2) enzymes [6].

Long-term NSAIDs therapy has been associated with an increased risk of gastric erosion and bleeding [7]. It has been estimated that about 60% of all patients chronically treated with NSAIDs present dyspeptic symptoms and around 30% have gastroduodenal ulcers [8,9]. On the other hand, despite the fact that this effect is decreased or abolished with selective COX-2 inhibitors, it has been reported that some patients undergoing chronic treatment demonstrated increased risk of adverse cardiovascular effects such as stroke and thrombosis [10].

**Figure 1:**
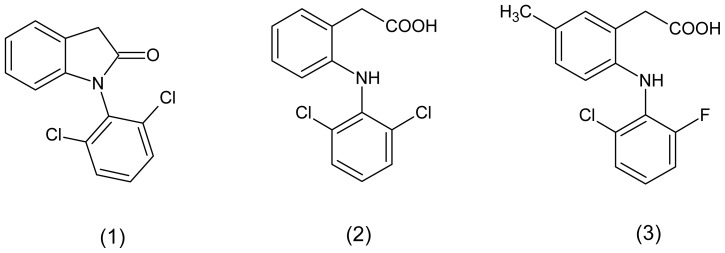
Chemical structures of diclofenac prodrug (**1**), diclofenac (**2**) and lumiracoxib (**3**).

Nowadays, the discovery of new drugs to treat chronic inflammation without adverse effects is one of the major challenges to pharmaceutical industry. Among the strategies useful to discovering new drugs, the molecular modification is a very promising strategy [11,12]. This approach could be useful to modify known NSAIDs in order to identify compounds with reduced gastrointestinal effects and different pharmacokinetic and pharmacodynamic profiles [13–15]. Using the prodrug approach, we have shown that lactam derivative (1) demonstrated anti-inflammatory and analgesic activity similar to the parental drug (Figure 1) [16].

As part of our ongoing program to identify new anti-inflammatory drug candidates with reduced gastric ulceration properties, we describe herein the anti-inflammatory pharmacological evaluation and the preliminary pharmacokinetics of the diclofenac prodrug, 1-(2,6-dichlorophenyl indolin-2-one (1), useful to treat chronic inflammatory conditions.

## 2. Results and Discussion

Diclofenac (2) was first introduced to the Japanese market in 1974. Nowadays, the drug is approved in 120 countries and is one of the most used NSAIDs worldwide. Despite the beneficial effects associated with decreased prostaglandin, thromboxan and leukotrienes levels, several studies have demonstrated that long-term therapy is associated with gastrointestinal irritation, ulceration and bleeding. The nonadhesion therapy’s adverse effects implicates many complications in chronic inflammatory diseases, including pain [17].

The gastro-toxic effect has been associated with two main factors: (a) inhibition of physiological prostaglandin synthesis in the stomach and; (b) local irritation caused by acid function of NSAIDs chemical structures. Several studies have demonstrated that molecular modification strategies could decrease this gastro-irritant effect by masking the carboxylic acid function [17–20]. Tammara and co-workers demonstrated that drug latention is a promising strategy to decrease the gastro-ulceronic effect. The authors’ synthesized esters prodrugs of diclofenac was less ulcerogenic and with 2000-fold increase in solubility over diclofenac [21]. Another study described the synthesis of diclofenac prodrugs as more lipophilic than parental drug with reduced gastro-ulcerogenic effects [22]. We have previously described that masking acid function of diclofenac into lactam derivatives could provide compounds with anti-inflammatory activity with the same nonulcerogenic properties. However, previous *in vitro* chemical and plasmatic studies have not demonstrated the conversion of prodrug to diclofenac [16,23].

### 2.1. Carrageenan-Induced Paw Edema

**Figure 2:**
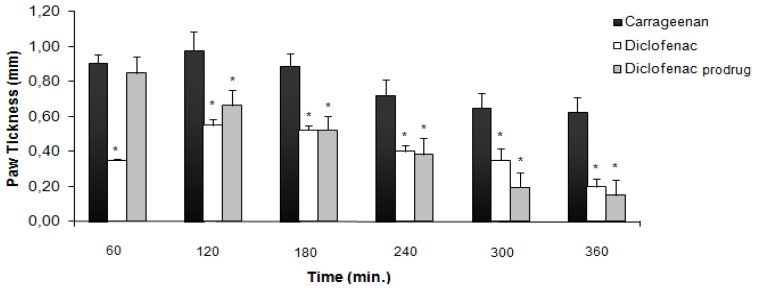
Anti-inflammatory activity using carrageenan-induced paw edema models in rats (data are represented as mean ± EPM, *n* = 6, *p < 0.05 with respect to carrageenan; dose of compounds = 300 μmol/kg).

Inhibition of swelling in carrageenan-induced edema in rat paw after oral administration of diclofenac prodrug (1) and diclofenac (2) at 300 μmol/kg is shown in Figure 2[24]. After 60 min, diclofenac (2) was able to reduce inflammation induced by carrageenan while the prodrug did not demonstrate activity. The prodrug (1) anti-inflammatory activity was comparable with diclofenac (2) after 180 min. All results were statistically significant when compared to the aqueous solution of sodium carboxymethylcellulose (0.5% *w*/*v*) used as the control (not shown). At 300 μmol/kg, the anti-inflammatory activity was the same observed in our previous at 100 μmol/kg [16].

### 2.2. Ulcerogenicity

The stomach ulcerogenicity caused by diclofenac prodrug (1), diclofenac (2) and lumiracoxib (3) at 300 μmol kg^−1^ was evaluated in order to study the gastro-irritant effect of these compounds (Table 1).

Acute administration of lumiracoxib (3) was able to induce an average of 19 gastric lesions, with punctual lesions (<1 mm) representing 89% of total lesions. According with methodology proposed by Cioli and co-workers, this kind of lesion could be classified as score two, because they have less than five small ulcers (1–2 mm) [25]. Diclofenac (2) induced at 300 μmol kg^−1^ an average of 94 lesions, with eight lesions greater than 2 mm. This result allows classifying this NSAID as score five. Interestingly, the diclofenac prodrug (1) demonstrated the ability to induce only punctual lesions. This prodrug is scoring as zero due the lesser ability to induce ulcer formation. We have observed that the number of ulcers at 300 μmol/kg was higher than at 100 μmol/kg. However, in both cases, the diclofenac prodrug (1) demonstrated to be safer than diclofenac (2) [16].

### 2.3. Effect of Diclofenac Prodrug Treatment on Carrageenan-Induced Release of PGE_2_

**Figure 3:**
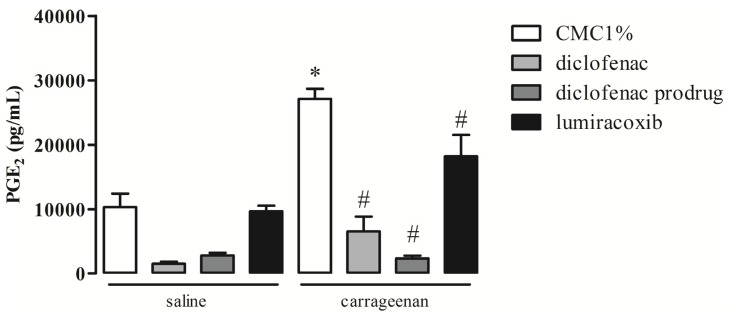
Effect of diclofenac prodrug (1) on PGE_2_ release in carrageenan-induced peritonitis. Groups of mice received diclofenac prodrug (1), diclofenac (2) or lumiracoxib (3) (14 mg/kg) or CMC1% (control) per os 1 h after cg or saline (control) i.p. injection. PGE_2_ was quantified in peritoneal exudates collected 4 h after cg or saline i.p. administration. Values are mean ± EPM of 4–8 mice. * *p* <0.05 when compared with the corresponding control group. # *p* <0.05 when compared with the cg group (positive control).

Production of PGE_2_ was measured by enzyme immunosorbent assay (EIA) in peritoneal washes collected 4 hr after intraperitoneal (i.p.) injection of carrageenan (cg) in mice. Animals were treated *per os* (p.o.) with diclofenac prodrug or NSAIDs (diclofenac or lumiracoxib—positive pharmacological controls), 14 mg/kg, or CMC 1% (positive control) 1 hr after cg i.p. injection. As we have observed in Figure 3, animals treated with CMC 1% p.o., 1 h after i.p. injection of cg, showed increased levels of PGE_2_ in peritoneal washes (27,125 ± 1574 pg/mL ), in comparison with those collected from animals without the cg stimulus (vehicle: 10,302 ± 2114 pg/mL). Oral treatment of animals with diclofenac prodrug 1 h after cg i.p. injection abolished cg-induced release of PGE_2_ (2345 ± 425 pg/mL) when compared with animals treated with CMC 1% 1 hr after i.p. injection of cg (positive control). Oral administration of diclofenac (pharmacological control) also abolished cg-induced PGE_2_ release (6554 ± 2305 pg/mL) when compared with the animals that received CMC 1% p.o. 1 h after cg i.p. In turn, the administration of lumiracoxib p.o. 1 h after cg i.p. injection promoted significant decrease in the PGE_2_ levels (18,173 ± 3345 pg/mL, 30% decrease) when compared with positive control.

Our results showed that treatment of mice with the new synthesized diclofenac prodrug (1) *per os* 1 h after an inflammatory stimulus markedly reduced major inflammatory events. By using an *in vivo* standard experimental model of inflammation, we showed that diclofenac prodrug (1) was able to abrogate the release of PGE_2_ and reduce leukocyte infiltration into the site of inflammation.

PGE_2_ is a lipid mediator which could increase in blood flow, edema formation and pain sensitization [26–28]. The inhibitory effect of diclofenac prodrug (1) was similar to diclofenac (2) and more potent than lumiracoxib. The effect of diclofenac (2), a nonselective COX-inhibitor, was also more effective than lumiracoxib, a selective COX-2 inhibitor. These results are in agreement with literature which demonstrated that PGE_2_ is produced mainly by COX-1in the first phase (1–4 h) of cg stimuli, while COX-2-derived PGE_2_ is observed in a second phase (6 until 96 h) [29]. However, in the present experimental condition, cg was able to induce COX-2 protein expression 4 h after its injection, suggesting that this isoform may be active at this period of time. Our results showed that lumiracoxib, a selective COX-2 inhibitor, promoted 30% reduction on PGE_2_ release induced by cg. Therefore, our data support the contention that PGE_2_ production in the present experimental condition is driven by the activities of both COX-1 and COX-2, being COX-1 the major isoform.

### 2.4. Effect of Diclofenac Prodrug Treatment on Carrageenan-Induced COX-2 Protein Expression by Peritoneal Leukocytes

**Figure 4:**
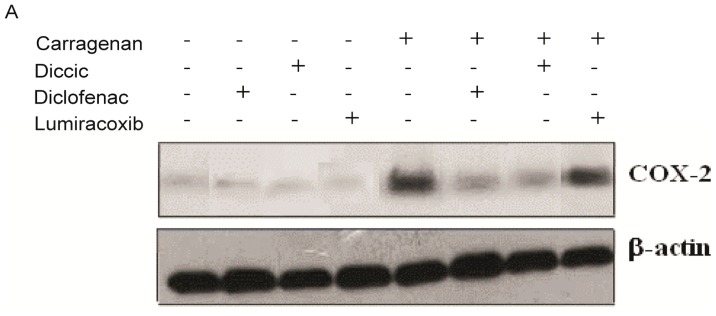

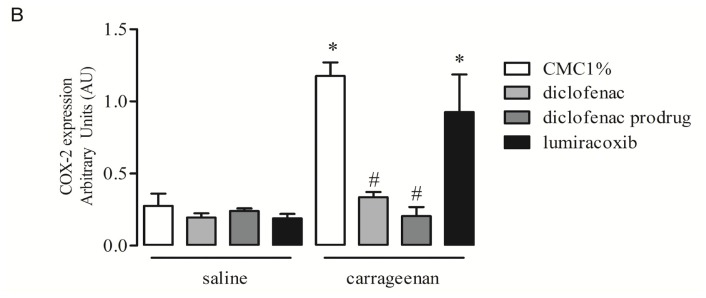
Effect dic-cic on carrageenan-induced COX-2 expression in peritoneal leukocytes. Groups of mice received diclofenac prodrug or diclofenac or lumiracoxib (14 mg/kg) or CMC1% (control) by oral administration 1 h after (**A** and **B**) cg (0.3% *w*/*v*) or saline (control) i.p. injection into the peritoneal cavity. Peritoneal leukocytes were collected 4 hr after i.p. administration of either cg or saline and whole cells were analyzed for COX-2 expression by Western blotting, as described in Materials and Methods. (**A**) Western blotting of COX-2, and β-actin (loading control) of leukocytes present in the inflammatory exudates; (**B**) Bar graph shows densitometric analysis of protein COX-2. The densities (in Arbitrary Units) were normalized with that of β-actin. Results were expressed as mean ± EPM from 5 to 8 mice. # *p* < 0.05 when compared with the positive control. * *p* < 0.05 when compared with the negative control group.

The effects of diclofenac prodrug (1) or NSAIDs (diclofenac (2) or lumiracoxib (3)—pharmacological controls), 14 mg/kg, or CMC 1% (positive control) on cg-induced COX-2 protein expression were measured in peritoneal leukocytes by Western blotting. As shown in Figure 4, the i.p. injection of cg (0.3% *w*/*v*) in animals treated with vehicle p.o.-induced COX-2 protein expression when compared with animals that received i.p. injection of saline and treated with vehicle p.o. (negative control). Oral administration of diclofenac prodrug (1) 1 h after cg i.p. injection, significantly downregulated COX-2 expression, when compared with animals treated with vehicle p.o. 1 h after cg i.p. injection (positive control). Administration of diclofenac (2) p.o. (pharmacological control) abolished cg-induced COX-2 protein expression. However, lumiracoxib oral treatment did not affect cg-induced COX-2 protein expression.

Diclofenac prodrug (1) abolished COX-2 protein expression suggesting that this effect contributes to the anti inflammatory effects of this new compound. Although NSAIDs are known to modulate prostaglandin production by inhibiting cyclooxygenases enzyme activities (COX), there are evidences that they have additional mechanisms of action [30]. Distinct signal transduction pathways, which involve COX-independent mechanisms, are already described for a number of selective and nonselective NSAIDs [31–33]. Several lines of evidence have shown in both *in vivo* and *in vitro* models that inhibition of some inflammatory cytokines or inhibition of NF-κB, and activation of PPAR-γ can promote inhibition of COX-2, explaining some regulatory effects of diclofenac on COX-2 pathway [32–38]. Since the diclofenac prodrug was based on diclofenac chemical structure, we hypothesized that this new compound may have mechanisms of action not dependent on COX inhibition, in close similarity to those of the progenitor molecule. On the other hand, our data showed that lumiracoxib did not inhibit COX-2 expression. These results are in accordance with Niederberger and co-workers, who demonstrated that lumiracoxib is able to inhibit COX-2 enzymatic activity, but does not able to inhibit COX-2 expression [39].

### 2.5. Effect of Diclofenac Prodrug Treatment on Carrageenan-Induced Cellular Influx into Peritoneal Cavity

**Figure 5:**
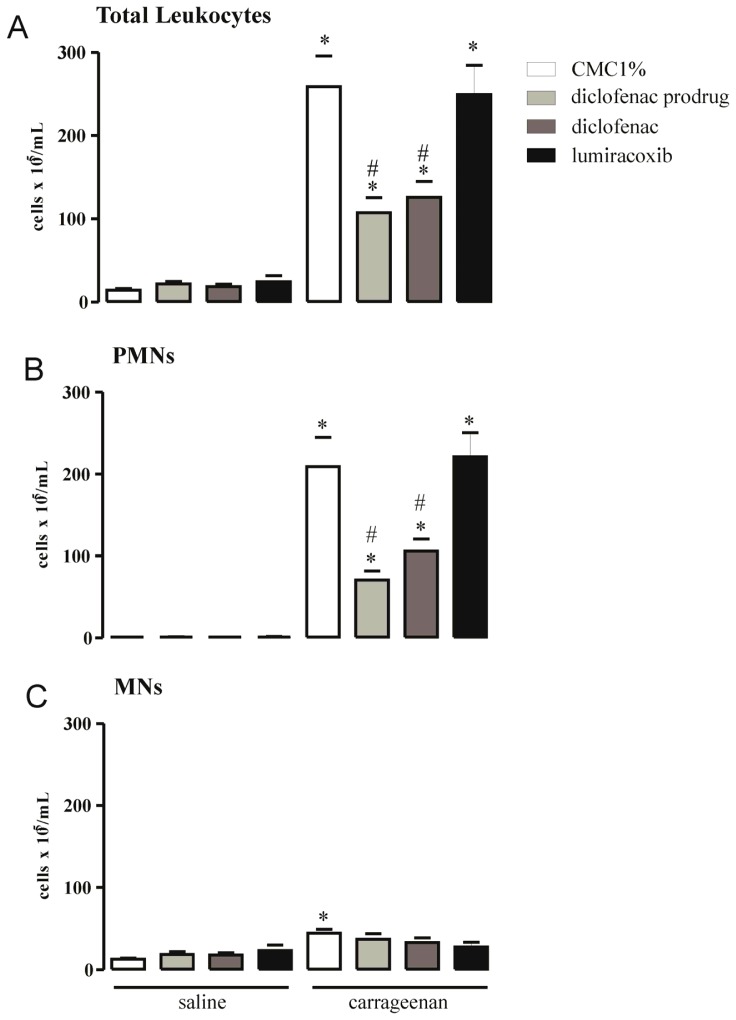
Effect of dic-cic on carrageenan-induced leukocyte influx into peritoneal cavity. Groups of mice received diclofenac prodrug or diclofenac or lumiracoxib (14 mg/kg) or CMC1% (control) by oral administration 1 h after (**A**–**C**) cg (0.3% *w*/*v*) or saline (control) i.p. injection. Total leukocyte (**A**), PMN (**B**) and MN (**C**) cell counts were determined in peritoneal washes harvested 4 h after cg or saline i.p. injection, as described in Material and Methods. Values are mean ± EPM of 4–8 animals. * *p* < 0.05 when compared with the corresponding control group. # *p* < 0.05 when compared with the group that received CMC 1% orally and cg i.p. injection (positive control group).

The effects of diclofenac prodrug (14 mg/kg) or CMC 1% (positive control) or NSAIDs (diclofenac or lumiracoxib—pharmacological controls) on cg-induced leukocyte accumulation in peritoneal cavities of mice were evaluated by total and differential cell counts in peritoneal washes collected 4 h after cg (0.3% *w*/*v*) i.p. injection. Figure 5A demonstrates a significant increase in total leukocyte numbers (258.6 ± 37.3 × 10^5^ cells/mL) in peritoneal washes collected from animals treated with CMC 1% p.o. 1 h after i.p. injection of cg (positive control) in comparison with washes obtained from animals that received CMC 1% p.o. and saline i.p. (14.3 ± 1.71 × 10^5^ cells/ml) (negative control). Oral administration of diclofenac prodrug 1 hr after the i.p. injection of cg significantly decreased the number of total leukocytes (107.4 ± 17.8 × 10^5^ cells/mL, 58% decrease) in peritoneal washes (Figure 5A), when compared with those from positive control. Diclofenac treatment promoted a significant reduction in the number of leukocytes in peritoneal washes (125.8 ± 19.2 × 10^5^ cells/mL, 51% decrease) in comparison with those collected from the positive group. Treatment of animals with lumiracoxib, p.o., did not modify cg-induced increase in the number of peritoneal leukocytes (249.2 ± 35.3 × 10^5^ cells/mL) (Figure 5A).

Differential cell counts showed that predominant leukocytes in peritoneal exudates of animals treated with CMC 1% 1 h after cg i.p. injection (positive control) were polymorphonuclear cells (PMNs) (209.1 ± 35.6 × 10^5^ cells/mL) followed by a low, but significant, number of mononuclear cells (MNs) (44.3 ± 4.9 × 10^5^ cells/mL) (Figure 5B,C). Numbers of PMNs and MNs in this group were significantly higher than those observed in peritoneal washes collected from the negative control group, which received CMC 1% p.o. 1 h after saline alone i.p. (PMNs: 0.9 ± 0.2 × 10^5^ cells/mL and MNs: 12.9 ± 1.5 × 10^5^ cells/mL). The number of PMNs in peritoneal fluid of mice was significantly reduced when animals were treated p.o. with diclofenac prodrug 1 h after the i.p. injection of cg (70.7 ± 11.1 × 10^5^ cells/mL, 66% decrease) compared with that of positive control group (Figure 5B). Diclofenac prodrug treatment did not change MNs numbers (36.8 ± 6.9 × 10^5^ cells/mL) in the same experimental condition (Figure 5C). Oral administration of diclofenac (pharmacological control) significantly decrease the cg-induced PMNs accumulation in peritoneal washes (106 ± 14.7 × 10^5^ cells/mL, 49% decrease), but did not alter the number of MNs (32.8 ± 5.9 × 10^5^ cells/mL). Treatment of animals with lumiracoxib (pharmacological control) neither altered the number of PMNs (221.3 ± 29.0 × 10^5^ cells/mL) nor the number of MNs (27.7 ± 5.5 × 10^5^ cells/mL) in washes from animals injected with cg (Figure 5B,C).

It was already established that intraperitoneal administration of cg produces a marked leukocyte influx into the local of its injection, being neutrophils mobilized within 1 h and then gradually replaced by monocytes/macrophages [40]. The diclofenac prodrug was able to inhibit cellular influx into peritoneal cavity induced by carrageenan, mainly PMNs. This effect was similar to that of diclofenac but different of lumiracoxib. Since cg-induced leukocyte influx is mediated by cytokines, adhesion molecules and mediators derived from arachidonic acid metabolism it is suggested that this diclofenac prodrug inhibitory activity is due to inhibition of chemotactic COX-derived eicosanoids, similarly to diclofenac action [41–45]. In regards to lumiracoxib, our results are in agreement with those of Cardoso *et al.* demonstrating that lumiracoxib did not alter neutrophil migration in a model of zymozan-induced PMN influx in rat [46].

### 2.6. Preliminary Plasma Concentration-Time Profile

**Figure 6:**
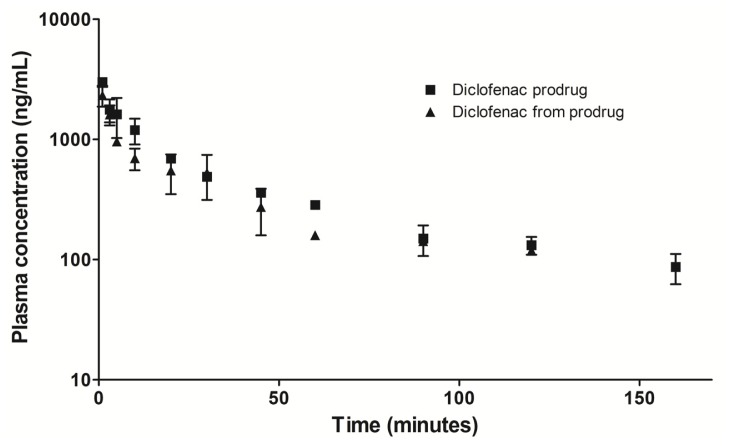
Plasma concentration *versus* time. Concentrations of diclofenac prodrug and diclofenac formed from prodrug. Diclofenac prodrug *i.v*. at 7.6 mg/kg using Wistar rats. Results are expressed as the mean ± SD (*n* = 6, three each time).

The plasma concentration-time curves of diclofenac prodrug and diclofenac formed from prodrug were demonstrated in Figure 6. After one minute of prodrug administration, diclofenac derivates from prodrug was quantified indicating rapid bioconversion. The concentration of diclofenac decays following prodrug plasmatic concentrations.

Preliminar *in vivo* pharmacokinetic study demonstrated bioconversion of diclofenac prodrug to parental drug after administration, despite *in vitro* chemical and plasmatic studies have not demonstrated this conversion [16]. These results allow us to corroborate the success of our molecular modification strategy.

## 3. Experimental Section

### 3.1. Material

All chemical reactives and solvents were commercial products of analytical or high performance liquid chromatography (HPLC) grades and they were purchased from Sigma–Aldrich Canada, Ltd., (Oakville, ON, Canada). The diclofenac prodrugs demonstrated purity of 98% and it was prepared according previously described [16].

### 3.2. Animals

Male Swiss mice (18–20 g) and male Wistar rats (200–250 g), were acclimated to housing for at least one week prior to investigation. The night before the experiment, food was withdrawn from the cages, but water as given *ad libitum*. Animals were randomly assigned to each treatment group and all testing was performed between 8:00 and 9:00 a.m. The research protocols and animals used were in accordance with the guidelines of the Committee for Ethics in the Use of Animals at the Butantan Institute, SP, Brazil (CEUAIB, reference number 614/09), CEUA-FCFAR (process 27/2006; process 27/2009) and international law and policies. All efforts were made to minimize the number of animals used and their suffering.

### 3.3. Carrageenan-Induced Paw Edema

The anti-inflammatory activity was evaluated using carrageenan-induced paw edema on rat method [24]. To evaluate the anti-inflammatory activity of the conjugates, four groups (*n* = 6) of Wistar rats (150–200 g) were examined. Group I served as a control group without using any drug, group II received diclofenac (2) at 300 μmol kg^−1^, and group III received diclofenac prodrug (1) at 300 μmol kg^−1^ as a homogeneous suspension in an aqueous solution of sodium carboxymethylcellulose (0.5% *w*/*v*), where the dose was molecularly equivalent to diclofenac. Each animal received 0.75–1.0 mL orally of the respective drugs. Thirty minutes after the administration of the drugs, each rat received a subplantar injection of 0.1 mL of 1% carrageenan solution in its left hind paw. The swelling volume of the paw was measured before (time 0) and at 60, 120, 180, 240, 300 and 360 min after the carrageenan injection. The measurement of the hind paw volume was carried out using an Ugo Basile Plethysmometer before any treatment (*V*_o_) and in any interval (*V*_t_) after the administration of the drugs. The percentage increase in the paw volume was calculated from the normal paw volume. The percentage of swelling inhibition was calculated using:

$$\%\;\text{inhibition}=[(V_{\text{t}}-V_{\text{o}})\text{control}-(V_{\text{t}}-V_{\text{o}})\text{treated}/(V_{\text{t}}-V_{\text{o}})]\times 100$$

where *V*_t_ and *V*_o_ relates to the average volume in the hind paw of the rats (*n* = 6) before any treatment and after anti-inflammatory agent treatment, respectively.

All the results are expressed as mean ± S.E.M. Statistical evaluation was performed using analysis of variance followed by Dunnet’s *t*-test for sub group comparison. A *p* < 0.001 was considered significant.

### 3.4. Ulcerogenicity

Gastrointestinal toxicity was determined using the method prevously described [25]. The studies were carried out on healthy Wistar rats (150–200 g) at 300 μmol kg^−1^. The animals were divided into four groups of six animals each, group I served as a control and received vehicle only. Group II received pure diclofenac at 300 μmol kg^−1^. Group III received diclofenac prodrug at 300 μmol kg^−1^. Group IV received lumiracoxib at 300 μmol kg^−1^. The animals were fasted 8 hr prior to a single dose of either the control or test compounds, given free access to food and water and sacrificed 17 h later. The gastric mucosa of the rats was examined using a 4× binocular magnifier. The lesions were counted and divided into large (greater than 2 mm in diameter), small (1–2 mm) and puntiform (less than 1 mm). For each stomach, the severity of mucosal damage was assessed according to the following scoring system: 0—no lesions or up to five puntiform lesions; 1—more than five puntiform lesions; 2—one to five small ulcers; 3—more than five small ulcers or one large ulcer; 4—more than one large ulcer. The mean score of each treated group minus the mean score of the control group was considered as the “severity index” of gastric damage. Statistical analysis was performed with analysis of variance (ANOVA) followed by Tukey’s test.

### 3.5. Treatments with Diclofenac Prodrug and NSAIDs

Groups of animals were deprived of food overnight and then treated *per os* (p.o.) with 200 μL of carboxy methyl cellulose (CMC 1%, *w*/*v*) (control group) or diclofenac prodrug dissolved in CMC1% (14 mg/kg). Other groups of animals were treated (p.o.) with either diclofenac (nonselective COX inhibitor) or lumiracoxib (selective inhibitor of COX-2) (14 mg/kg, p.o.), both dissolved in CMC1%. These groups of animals were used as pharmacological controls. All oral treatments were performed 1 h after cg i.p. injection.

### 3.6. Carrageenan-Induced Inflammation in Peritoneum of Mice

Animals received intraperitoneal (i.p.) injection of carrageenan (cg) dissolved in sterile saline (0.3%, *m*/*v*) in a volume of 1 mL. Another group of animals received i.p. injection of 1 mL sterile saline as negative control of inflammation. Animals were killed by over-exposure to CO_2_ 4 h after cg or saline i.p. injection, and the peritoneal exudates were withdrawn after washing the peritoneal cavities with 2 mL of saline solution. Aliquots of washes were then used for analysis of selected inflammatory events (leukocyte infiltration, COX-2 protein expression by leukocytes and PGE_2_ release into peritoneum).

### 3.7. Leukocyte Harvesting and Counting

Aliquots of the peritoneal washes were used to determine total cell counts in a Newbauer chamber after dilution (1:20, *v:v*) in Turk’s solution (0.2% crystal violet dye in 30% acetic acid). For differential cell counts, cytospin preparations were stained with Hema3 stain. Differential cell counts were performed by counting at least 100 cells, which were classified as either polymorphonuclear (PMNs) or mononuclear (MNs) cells, based on conventional morphological criteria. In addition, a volume corresponding to 2 × 10^6^ cells was centrifuged at 500 g for 6 min at 22 °C. Cell pellet was used to analyze COX-2 expression by Western blotting and supernatant used for PGE_2_ quantification by enzyme immunoassay (EIA).

### 3.8. Western Blotting

Obtained cells (2 × 10^6^) were lysed with 100 μL of sample buffer (20% SDS; glycerin; 1 M mercaptoethanol; 0.5 M Tris buffer pH 6.8; 0.1%bromophenol blue) and heated at 100 °C for 10 min [47]. An aliquot of 14 μL of the lysate was separated on SDS-polyacrilamide gel (10%) at 150 V and eletrophoretically transferred to a nitrocellulose membrane (GE Healthycare, Buckinghamshire, UK). The membrane was blocked with 5% nonfat milk in Tris-buffered saline with 0.05% Tween 20 and incubated 1 h at room temperature with the antibody against COX-2 (1:1500) (Cayman Chemicals, Ann Arbor, MI, USA) followed by incubation in the same buffer with the appropriate antirabbit horse hadish peroxidase-conjugated secondary antibody (GE Healthycare, Buckinghamshire, UK) for 1 hr at room temperature (1:1500). Further, the membrane was incubated with the antibody against β-actin (1:2000) (Sigma, St. Louis, MO, USA) followed by incubation with the antimouse secondary horse hadish peroxidase-conjugated antibody (1:2000) (GE Healthycare, Buckinghamshire, UK). Immunoreactive bands were detected using ECL kit (GE Healthycare, Buckinghamshire, UK). Densities of the bands were determined by a GS 800 Densitometer (Bio Rad Laboratories, Richmond, CA, USA) using the image analysis software from Molecular Analyst® (Bio Rad Laboratories, Richmond, CA, USA).

### 3.9. PGE_2_ Quantification

Concentrations of PGE_2_ were determined by specific enzyme immunoassay using a commercial kit (Cayman Chemical Company, Ann Arbor, MI, USA) [48]. The extraction of PGE_2_ was performed on Sep Pak C18 columns (Waters Corporation, Milford, MA, UK) and eluted with ethanol. In brief, 50 μL aliquots of each extracted sample were incubated with the PGE_2_ conjugated with acetylcholinesterase and the specific rabbit antiserum in 96-well plates, coated with antirabbit IgG mouse monoclonal antibody. After addition of the substrate, the absorbance of the samples was recorded at 405 nm in a microplate reader (Thermo-Labsystem Multiscan®, Helsinki, Finland), and concentrations of PGE_2_ were estimated from standard curves.

### 3.10. Data Analysis

Results are expressed as mean ± SEM. Differences between groups were analyzed by one-way ANOVA, followed by the Tukey test. Differences with an associated probability (*p* value) of less than 5% (*p* < 0.05) were considered significant.

### 3.11. Preliminary Plasma Concentration-Time Profile

A plasma concentration-time profile of diclofenac prodrug and diclofenac from prodrug administration were performed to observe the diclofenac from prodrug formation. This assay was carried out using Wistar rats (*n* = 6, three for each time). The animals were subjected to catheter implantation in the femoral artery and vein. Diclofenac prodrug (7.6 mg/kg) was administered in the femoral vein catheter. Blood samples (0.5 mL, each) were collected into heparinized tubes *via* femoral artery by the implanted catheter. The blood was centrifuged at 2500 rpm for 10 min, to separate the plasma until HPLC analysis.

## 4. Conclusions

The diclofenac prodrug (1) exhibits *in vivo* anti-inflammatory activity, using carrageenan-induced paw edema at 300 μM, without gastro ulcerogenic effect. The prodrug was able to decrease PGE2 levels, COX-2 expression and cellular influx into peritoneal cavity induced by carrageenan treatment. Preliminar pharmacokinetic studies demonstrated that prodrug is bioconverted *in vivo* to diclofenac. The ability of diclofenac prodrug (1) to inhibit inflammatory events when administered after inflammatory stimuli makes this compound a promising new anti-inflammatory drug that should be further investigated and developed for future therapeutic use.

## Figures and Tables

**Table 1:** Ulcerogenic effect of diclofenac prodrug (1), diclofenac (2) and lumiracoxib (3) in rats (*n* = 6, mean ± SD).

Compounds	Number of ulcers	<1 mm	1–2 mm	>2 mm

Lumiracoxib	19 ± 5.1 *	17 ± 4.2 (89%)	2 ± 0.5 (11%)	0
Diclofenac	94 ± 12.8	66 ± 8.2 (70%)	20 ± 2.8 (29.9%)	8 ± 3.2 (0.1%)
Diclofenac prodrug	3 ± 1.1 *	3 ± 1.1 (100%)	0	0

* Significant difference compared to group that received diclofenac. *p* < 0.05 (Tukey’s test).
